# Risk of HPV-related extra-cervical cancers in women treated for cervical intraepithelial neoplasia

**DOI:** 10.1186/s12885-020-07452-6

**Published:** 2020-10-07

**Authors:** Mario Preti, Stefano Rosso, Leonardo Micheletti, Carola Libero, Irene Sobrato, Livia Giordano, Paola Busso, Niccolò Gallio, Stefano Cosma, Federica Bevilacqua, Chiara Benedetto

**Affiliations:** 1 Department of Surgical Sciences University of Torino, Via Ventimiglia 3, 10126 Torino, Italy; 2 Piedmont Cancer Registry – CPO, Torino, Italy; 3 Cancer Prevention Center of Piedmont, Torino, Italy

**Keywords:** HPV-related cancers, Anogenital area, Anal cancer, Vaginal cancer, Vulvar cancer, Multiple subsequent cancers

## Abstract

**Background:**

The aim was to estimate the risk of subsequent extra-cervical Human Papillomavirus (HPV) related cancer in patients surgically treated for high grade cervical intraepithelial neoplasia (CIN 2–3). This is the first study in Italy investigating the occurrence of extra-cervical tumors in this cohort of patients.

**Methods:**

3184 patients surgically treated for CIN2–3 since 1992 at the Department of Surgical Sciences of University of Torino were considered. The risk of HPV-related cancer was calculated as Standardized Incidence Ratio (SIR), using as expected values tumour age specific incidence of resident population.

**Results:**

173 second primary cancer (SCPs) were identified. SIR to develop cancer after treatment for CIN2–3 was 2.2 (CI 95% 1.89–2.50). Among these occurrences, 10 are in HPV related sites: 1 anus (SIR = 1.8; 0.04–10.0), 3 vagina (SIR = 12.4; 2.56–36.3), 1 vulva (SIR = 1.7; 0.04–9.59), 5 oropharynx (SIR = 8.5; 2.76–19.8).

Significant risk has been also recorded for pulmonary (SIR = 3.1; 0.70–5.27) and bladder (SIR = 4.05; 1.10–10.56), with smoking as possible cofactor. We also found increased risk for breast (SIR = 2.4; 2.07–2.84) and ovarian cancers (SIR = 2.1; 1.13–3.49), probably due to an higher adherence to spontaneous and programmed screening programs.

**Conclusions:**

Our study supports the hypothesis of an increased risk of HPV-related tumours for CIN treated patients, mostly for CIN3. It is conceivable the need of early diagnosis for these cancers in this higher-risk populations.

## Background

High-risk human papillomaviruses (hrHPV) are estimated to cause 5.2% of all cancers worldwide, with HPV16 alone responsible for approximately 70% of cervical cancer, that is the first most common HPV-related cancer in women [1]. HPV are also detectable in 15–48% of vulvar, 68–86% of vaginal, 85–91% of anal,19–25% of oropharyngeal, 3–5% of oral cavity, 2–5% of larynx cancers [2–5].

Precancerous lesions precede these cancers, and cervical intraepithelial neoplasia (CIN) is the most studied and treated. Despite CIN treatment has a high impact in reducing cancer risk (from 95 to 99%), treated women are still considered at higher risk of cervical malignancies compared to the general population and at risk of HPV-related malignancies in other body sites [6, 7]. Many studies demonstrated that women with history of diagnosis and treatment for high-grade CIN are at increased risk for HPV subsequent primary cancers (SPCs) [6–16]. An HPV-SPC is defined as a metachronous invasive primary solid tumour, which develops at least 2 months after the diagnosis of a primary HPV-related tumour [17].

Viral, host, and behavioral variables may increase the risk of cancer development allowing persistence of HPV infection.

The viral genotype present influences risk and HPV 16 infection has the highest risk of viral persistence and subsequent precancer/cancer development [15].

Host immunoresponse influence the effectiveness of viral clearance: primary immunodeficiency disorders but also polymorphisms in genes such as TLRs (Toll-Like receptors) and NF-kB networks are associated with higher risk of lesions [18]. Also secondary immunodeficiencies either infection-related (HIV) or iatrogenic (chemotherapy or biological drugs) elevate the risk of HPV related malignancies [4].

Sexual behaviors such as number of partners, young age at first intercourse, frequency and type of sexual practices and partners’ sexual histories are risk factors for developing HPV related cancers [19]. Smoking is still debate as risk factors, but it seems that it can play a role in transition from HPV infection to precancer [20].

The aim of this study was to estimate the risk of developing SPCs and the site of occurrence in a cohort of women resident in Piedmont (North-West Italy Region) previously treated for high-grade CIN, distinguishing between HPV-related and non-HPV-related sites.

This is, at our knowledge, the first study in Italian population with the aim to provide accurate risk estimates of occurrence of a prior CIN represents an increased risk for HPV-related SPCs.

## Methods

Individual clinical information on all patients surgically treated with large loop excision of transformation zone (LLETZ) procedure at the Department of Surgical Sciences of the University of Torino, St. Anna Hospital for high-grade CIN from 1992 to 2016 were recruited in the cohort (6649 patients). Women treated after 2014 were excluded in order to have at least 5 years of follow-up for each patient, reducing the cohort at 5595 women. This database was linked with the Piedmont Cancer Registry (Registro Tumori Piemonte: RTP) to retrieve information on subsequent neoplasm (excluding non-melanoma skin cancers). RTP registered cancers among residents only in the Municipality of Turin between 1985 and 2007, the residents in the larger Turin Metropolitan Area since 2008, and finally in the whole region since 2013. Patients in the cohort were considered only if resident in the corresponding area for the period above mentioned, otherwise they were discarded from the cohort. The study cohort was thus reduced to 3184 patients.

Expected cases by type of cancer were calculated in the reduced cohort applying corresponding specific rates [http://ci5.iarc.fr; www.cpo.it] by age class (five-year groups: 0–85+), period (Turin Municipality: years 1992–1997, 1998–2002, 2003–2007, 2008–2012, 2013–2014; Metropolitan Area: years 2008–2012, 2013–2014; Piedmont Region: years 2013–2014) and cancer site. Person-years in the cohort were calculated with month approximation at follow-up date and time of surgery (as starting observation time).

All tumours were classified by anatomical site using the International Classification of Cancer Pathologies, 3rd edition ICD-O 3. Risk of SPCs was then calculated dividing number of observed and expected cases as Standardized Incidence Ratio (SIR). Confidence interval of SIR were derived using Haenszel’s exact method [21].

## Results

The study cohort of 3184 patients resident in Piedmont during the period and in the area of RTP registration was observed for a total of 20,022 person-years up to the end of 2014.

In this period, a total of 173 neoplasms were registered in these women (Table 1) with an overall SIR of 2.2 (95% exact CI: 1.89–2.50). In particular, HPV-related cancer site showed an important and statistically significant risk increase in patients surgically treated for CIN2–3. Overall oropharynx cancers (5 cases) exhibited a SIR of 8.5 (95% exact CI: 2.76–19.8). Tongue cancers were the most frequent among them, 4 occurrences and SIR of 14.1 (95% exact CI: 3.84–36.2).

**Table 1:** Observed subsequent cancers in a cohort of patients treated with LLETZ for CIN2–3 [excluding non-melanoma skin cancers]

Cancer Site	ICDO-3 codes	Observed SPCs
Oropharynx	C01-C10	5
Oesophagus	C15	1
Colon-rectum	C18–20	20
Anus	C21	1
Liver	C22	1
Gallbladder	C24	2
Pancreas	C25	1
Lung	C33-C34	15
Melanoma	C44	11
Soft Tissues	C49	1
Breast	C50	62
Vulva	C51	1
Vagina	C52	3
Corpus Uteri	C54	9
Ovary	C56	7
Kidney	C64	2
Bladder	C67	3
SNC	C71	1
Thyroid	C73	6
Parathyroid	C75	1
Myeloma	C42	5
Lymphomas	C77	9
Leukaemias	C42	2
Others		4
**Total**		**173**

Legenda:

*ICDO-3* International Classification of Diseases for Oncology 3rd Edition

*LLETZ* Large Loop Excision of the Transformation Zone

*SPCs* Second Primary Cancers

We had a SIR of 1.8 (95% exact CI: 0.04–10.00) in anal area, 1,7 (95% exact CI: 0.04–9.59) and 12.4 (95% exact CI: 2.56–36.3) in vulvar and vaginal localization respectively.

Summing up all these HPV related sites we had 10 cases with a significant SIR of 5.1 (95% exact CI: 2.44–9.38). For each positive case of SPC, we checked, consulting the database created, whether patients were treated for CIN 2 or CIN 3: an higher risk of developing SPCs was found in CIN 3 treated patients, SIR of 6.08 (95% exact CI: 2.44–12.53), compared to CIN2, SIR of 1.43 (95% exact CI: 0.57–2.94). (Table 2).

**Table 2:** HPV-related cancers in CIN 2–3 LLETZ treated patients. Standardized Incidence Rates according to Haenszel’s formula

Cancer Site	ICDO-3 codes	Observed SPCs	Expected SPCs	SIR	95% CI
Oropharynx	C01-C10	5	0.59	8.5	2.76–19.8
Anus	C21	1	0.55	1.8	0.04–10.0
Vulva	C51	1	0.58	1.7	0.04–9.59
Vagina	C52	3	0.24	12.4	2.56–36.3
**Total**		**10**	**1.96**	**5.1**	**2.44–9.38**

Legenda:

*ICDO-3* International Classification of Diseases for Oncology 3rd Edition

*LLETZ* Large Loop Excision of the Transformation Zone

*SPCs* Second Primary Cancers

*SIR* Standardized Incidence Ratio

*CI* Confidence Interval

An excess risk of other cancers not strictly related to HPV infection was also observed. In particular, 15 lung cancers, SIR 3.1 (95% exact CI: 0.70–5.27); 3 bladder cancers, SIR 4.05 (95% exact CI: 1.10–10.56). 62 subsequent breast cancers and 7 ovarian cancers ((SIR respectively 2.4 (95% exact CI: 2.07–2.84) and 2.1 (95% exact CI: 1.13–3.49)).

## Discussion

The study was performed to estimate the risk of SPCs in extra-cervical sites in women surgically treated for CIN 2 and CIN 3. Recently, similar studies have been published, with slightly different criteria of inclusion [6–14, 16, 22–24]. Sexually transmitted HPV infection is common, but usually transient: 80% of infected women spontaneously eliminate HPV within 2 years of acquisition [3].

The presence of high-grade CIN is a good proxy for persistent high-risk HPV infection. The common viral HPV aetiology for a subset of extra cervical tumours factor explains why women with history of CIN2 or CIN3 have a significantly higher risk of anal, vulvar, vaginal, and oral cancer [16, 25].

Women whose inadequate immune response failed to clear HPV infection and allowed viral persistence and CIN development are at higher risk for HPV persistence after CIN treatment and subsequent related lesions [24].

An immunological failure could be present, together with other specific risks, in other body sites, consequently increasing the risk of lesions elsewhere [4, 17]. The common risk factors as HPV 16 persistent infection and other behavioural factors as smoking and sexual practices could also increase risk for SPCs development, in the presence of a persistent HPV infection [3].

### Anogenital region

In (Table 3) we compare our study with the literature data. All studies use SIR or Relative Risk (RR) to estimate the correlation between primary cervical lesions and SPCs.

**Table 3:** Anogenital SPCs in CIN 2–3 LLETZ treated women. A comparison between literature and our study data

Author	Year	First lesion	Value	Vulva	Vagina	Anus
Evans [26]	2003	CIN 3	SIR	4.4	18.5	5.9
Kalliala [6]	2005	CIN 1–3	SIR	4.1	12	5.7
Edgren [9]	2007	CIN 3	IRR	2.2	6.7	4.7
Strander [22]	2007	CIN 3	RR		6.8	
Saleem [12]	2007	CIN3	SIR	–	–	16.4
		SCC	SIR	–	–	6.2
Tatti [27]	2012	CIN 2–3	OR	–	–	1.91
Gaudet [10]	2014	CIN 2	SIR	1.47	3.61	0.89
		CIN 3	SIR	3.79	8.53	2.28
		CIN2–3	SIR	2.9	6.65	1.75
Sand [11]	2016	CIN 2	RR	2.5	8.1	2.9
		CIN 3	RR	4	17.1	4.2
Ebisch [8]	2017	CIN 3	IRR	4.97	86.08	3.85
Suk [23]	2018	SCC	SIR	3.8	17.3	2.3
Acevedo Fontánez [14]	2018	SCC	SIR	–	–	51.6
Wang [16]	2020	SCC	SIR	2.29	1.85	1.41
Our study		CIN 2–3	SIR	1.7	12.4	1.8

Legenda:

*CIN* Cervical Intraepithelial Neoplasia

*IRR* Incidence Rate Ratio

*LLETZ* Large Loop Excision of the Transformation Zone

*RR* Relative Risk

*SCC* Invasive Squamous Cell Carcinoma

Surgical removal (LLETZ) of CIN does not mean removal of HPV infection and HPV DNA can be present in surrounding, clinical normal tissues and this viral persistence can rise the subsequent precancer/cancer risk [7].

The correlation between anogenital tumour or pre-tumour lesions and HPV infection has been well described [18]. There is general consensus in stating that patients treated for high grade CIN or cervical cancer are at increased risk of invasive neoplasia and our study confirms this trend [5–14, 16].

In our study all SPCs found in anal, vulvar and vaginal sites occurred in women with previous history of CIN 3. No case of CIN 2 determined SPCs in the anogenital area. This could be explained by the more reproducible diagnosis of CIN 3 respect to CIN 2 and with the longer HPV persistence to achieve CIN3/carcinoma in situ lesions [28]. Also HPV-induced field effect is a risk factor for multicentric disease, as a persistent lesion allows continuous spreading of infective HPV [29].

### Head-neck

The role of HPV in the pathogenesis of a subset of head and neck tumours, in particular in oropharyngeal squamous cell tumours (OPSCCs), has been well established [30, 31]. Patients with HPV-related cancers are more frequently younger and with a better prognosis than those with OPSCCs HPV-negative [31]. Numerous studies in the literature show an increased risk of SPCs onset after anogenital HPV-related lesion [6–8, 10, 23, 26]. We confirmed this evidence. (Table 4) compares the values of the literature with our study**.**

**Table 4:** Head and neck SPCs in CIN 2–3 LLETZ treated women. A comparison between literature and our study data

Author	Year	First lesion	Value	Head and Neck
Evans [26]	2003	CIN 3	SIR	1.2
Kalliala [6]	2005	CIN 1–3	SIR	1.7
Gaudet [10]	2014	CIN 2	SIR	0.47
		CIN 3	SIR	0.67
Ebisch [8]	2017	CIN 3	IRR	5.51
Suk [23]	2018	SCC	SIR	1.4
Wang [16]	2020	SCC	SIR	2.29
Our study		CIN 2–3	SIR	8.5

Legenda:

*CIN* Cervical Intraepithelial Neoplasia

*IRR* Incidence Rate Ratio

*LLETZ* Large Loop Excision of the Transformation Zone

*SCC* Invasive Squamous Cell Carcinoma

*SIR* Standardized Incidence Ratio

A Danish study shows that women with HPV-related OPSCC are more likely to be affected by cervical disease: the association between oral and cervical HPV related lesions is stronger for smoking patients than for non-smokers [30]. Also sexual behaviours as unprotected oral sex and number of lifetime partners are considered risk factors. With the exception of HPV16, the HPV types that infect the cervix are different from those of oral area, and carcinogenesis mechanisms are not identical, even not yet fully understood [31]. OPSCC arise in a different epithelium from the cervical one without transformation zone, and no preinvasive oral lesion can be identified up to date. HPV genome integration occurs with different patterns: in cervical cancer through the classic E2 breakpoint, while in OPSCC throughout the entire genome, more frequently in E1 [31].

Unfortunately, we could not perform stratification to smoking habit because this information was not systematically recorded in our database.

### Lung and bladder

There is recent hypothesis about the role of HPV in these cancers [32]. The most plausible cause of correlation between cervical and lung or bladder cancers is the common risk factor of smoking [32–34].

It has been hypothesized an association between bladder cancer and HPV. Anatomical proximity could be a potential cause for viral migration, since in women urethra is close to anogenital area which could be a reservoir for HPV. Also natural HPV tropism for squamous epithelium could facilitate infection [35].

As for lung, there is a variable reported incidence of HPV DNA in lung cancer specimens and it has been speculated that HPV reaches the lungs via the bloodstream, even if it is known that HPV does not cause generalized viremia [36]. It has been also proposed that HPV particles may be carried though air, raising the potential issue of occupational exposure for healthcare providers and exposure for patients during LLETZ procedures [37].

In our series all SPCs occurred after a CIN3 history, whereas in the lungs a greater heterogeneity of results was found: 9.8% after CIN1 history, 35.7% after CIN2 and 35.7% after CIN3. While 25% of bladder lesions occur after CIN2 history and 75% after treatment for CIN 3.

### Other Gynaecological sites

Some studies aimed to find a biologically and clinically plausible explanation for breast or ovarian tumour occurrences in patients with a history of HPV-related lesions. Despite these studies, the scientific evidences of HPV involvement in breast carcinogenesis is not sufficient yet [38, 39]. In breast and ovary numerous tumour occurrences have been found. We thought that this increased risk could depend on increased diagnostic attention and adherence to organized and spontaneous screening programs.

The role of hormones in tumorigenesis in breast, ovary and thyroid cancers is to be considered as a possible common risk factor.

## Conclusions

Cervical cancer screening program in Italy is constantly updated by GISCI, Italian Cervicocarcinoma Screening Group (www.gisci.it). Due to the low incidence of anal cancer (1–3 cases/100,000 women-years), vaginal cancer (0.5–1.7 cases/100,000 women-years) and vulvar cancer (1-to 4.6 cases/100,000 women-years) there are no cost-effective screening programs for these tumours [40–43].

As invasive tumour lesions in these sites are preceded by pre-tumoral lesions and non-invasive diagnostic examinations are available, it could be appropriate to consider women treated for CIN3, as at greater risk of other HPV-related cancers, to be target of screening strategies.

To plan appropriate program for cervical precancer and cancer detection it should be suggested to continue screening after the age that usually negative women stop (65 years in Italy), as risk continues to be elevated even after 20 years from first treatment [7].

This prolongation or even a lifelong cervical cancer screening will allow detection of vaginal, vulvar and anal precancerous lesions too. For vaginal cancer, the personalized follow-up program would benefit from the high sensitivity of validated HPV tests combined with the high specificity of the cytology and colposcopy. Vulvar preinvasive lesions could also be detected during routine examination before the insertion of vaginal speculum to perform HPV or PAP test, through the correct and conscious inspection of the external genitalia. Thus it is of paramount importance to train healthcare providers (general practitioners, gynecologists and midwives) to a general assessment of genitalia.

In anal region, screening in CIN3 treated patients could use a threefold approach: cytology, high-resolution anoscopy and guided biopsy as the best resource currently available [44]. However there are no validated approaches to anal cancer screening due to its low incidence and the best option is limiting screening to highest risk subgroup, such as immunodepressed women (either from HIV or iatrogenic immunosuppression), women with multiple sexual partners, heavy smokers and with history of anal warts [45].

Despite the high specificity (92%) and moderate sensitivity (72%) of oral HPV detection methods [oral rinsing and oral swabs], even for head and neck cancers the opportunity of a screening program is affected by the low incidence in the general population of the same, especially in women. Moreover, unlike tumours in the anogenital area, there is no pre-tumour lesion that anticipates oral and oropharyngeal cancers, this further compromises the usefulness of a screening strategy [30, 46].

It remains to assess the impact of adjuvant HPV vaccination in treated patients: it has already demonstrated a protective effect in reducing recurrent intraepithelial cervical lesion but further studies are needed to evaluate the impact on HPV-related SPCs [47].

There are some limitations in our study. First the type of treatment of cervical lesions, LLETZ, could have influenced the study outcome, since it removes less tissue than cold knife conization. If this approach is of utmost clinical benefit for reduction of adverse obstetrics outcomes, it can leave in situ more HPV infected tissue. Also positive margin status and glandular involvement from a less invasive treatment could have influenced, as a result of incomplete excision or a lesion hidden in the glandular crypts. Secondly, our database is not representative of the entire Italian population but based on a regional cancer registry. Furthermore, we assumed that SPCs can be attributable to HPV infection persistence but HPV testing was not available in our retrospective database.

The increased risk of cancer in extra-cervical sites after CIN 2–3 treatment makes appropriate to introduce personalized follow-up and screening programs, while HPV vaccination needs further studies to assess its impact. The best strategy should be defined according to the economic budget of public health and to the workload of the hospital units in charge of their implementation.

## Data Availability

The datasets used and/or analyzed during the current study are available from the corresponding author on reasonable request.

## Abbreviations

CIN: Cervical Intraepithelial Neoplasia
HPV: Human Papillomavirus
LLETZ: Large Loop Excision of the Transformation Zone
SIR: Standardized Incidence Ratio
SCP: Second Primary Cancer
RTP: Registro Tumori Piemonte, Piedmont Cancer Registry
OPSCC: Oropharyngeal Squamous Cell Tumours
RR: Relative Risk
